# Quantum entropy and central limit theorem

**DOI:** 10.1073/pnas.2304589120

**Published:** 2023-06-12

**Authors:** Kaifeng Bu, Weichen Gu, Arthur Jaffe

**Affiliations:** ^a^Department of Physics, Harvard University, Cambridge, MA 02138; ^b^Department of Mathematics and Statistics, University of New Hampshire, Durham, NH, 03824; ^c^Department of Mathematics, Harvard University, Cambridge, MA 02138

**Keywords:** convolution, entropy, central limit theorem

## Abstract

We introduce a framework to study discrete-variable (DV) quantum systems based on qudits. It relies on notions of a mean state (MS), a minimal stabilizer-projection state (MSPS), and a new convolution. Some interesting consequences are: The MS is the closest MSPS to a given state with respect to the relative entropy; the MS is extremal with respect to the von Neumann entropy, demonstrating a “maximal entropy principle in DV systems.” We obtain a series of inequalities for quantum entropies and for Fisher information based on convolution, giving a “second law of thermodynamics for quantum convolutions.” We show that the convolution of two stabilizer states is a stabilizer state. We establish a central limit theorem, based on iterating the convolution of a zero-mean quantum state, and show this converges to its MS. The rate of convergence is characterized by the “magic gap,” which we define in terms of the support of the characteristic function of the state. We elaborate on two examples: the DV beam splitter and the DV amplifier.

## Significance

Convolution has a significant impact on many scientific disciplines, ranging from probability theory and harmonic analysis to information theory. Here, we introduce a framework to study quantum convolution in discrete-variable (DV) quantum systems. We establish a maximal entropy principle and a quantum central limit theorem for DV quantum systems. We also provide a bound on the rate of convergence in the central limit theorem that we call the magic gap. The magic gap has some similarities to the Cheeger constant in graph theory. Our convolutional framework provides an approach to study stabilizer and magic states.

Quantum information and quantum computation come in two forms, continuous-variable (CV) and discrete-variable (DV) systems. CV quantum information has been widely used in quantum optics and other settings to deal with continuous degrees of freedom (1). Gaussian states, and processes which can be represented in terms of a Gaussian distribution, are the primary tools used in studying CV quantum information. One important property of Gaussian states is their extremality within all CV states, under some constraint on the covariance matrix (2–6). Gaussian states also minimize the output entropy or maximize the achievable rate of communication by Gaussian channels. One sees this using quantum entropy–power inequalities on the convolution of CV states (7–17). This statement is a quantum analogue of Shannon’s entropy power inequality (18–20). These states have both been realized in experiment, and also applied in quantum information tasks, such as quantum teleportation (21–23), quantum-enhanced sensing (24–27), quantum-key distribution (28), and quantum-speed limits (29).

However, computational processes with only Gaussian states and processes can be efficiently simulated on a classical computer (30–32). Hence, non-Gaussian states and processes are necessary to implement universal quantum computing (33, 34). To quantify the non-Gaussian nature of a quantum state or process, the framework of resource theory has been used (35–37). CV quantum systems have also been considered as a platform to implement quantum computation and realize quantum advantage. Several sampling tasks have been proposed (38–41), including Gaussian boson sampling, a modification of the original boson sampling proposed by Aaronson and Arkhipov (42). This has attracted much attention and has been realized experimentally; it is claimed that they display a quantum advantage over classical computers (43–45).

This raises a natural question, “what states in DV quantum systems play the role of Gaussian states in CV quantum systems?” Here, we focus on stabilizer states. They are the common eigenstates of certain abelian subgroups of the qubit Pauli group and were introduced by Gottesman to study error correction (46). There are several indications that stabilizer states are the finite-dimensional analogue of Gaussian states in CV quantum systems. For example, the Hudson theorem for CV systems states that the Wigner function of a pure state is nonnegative, if and only if the state is Gaussian (47, 48). On the other hand, Gross proved in DV systems with the local dimension being an odd prime number, that the discrete Wigner function of a pure state is nonnegative, if and only if the state is a stabilizer (49).

From the Gottesman–Knill theorem (50), we infer that stabilizer circuits comprising Clifford unitaries with stabilizer inputs and measurements can be efficiently simulated on a classical computer. In fault-tolerant quantum computation, logical Clifford unitaries can be implemented transversally so they are considered to be low-cost. However, the Eastin–Knill theorem (51) states that there is no quantum error correction code in which any universal gate set can be implemented transversally. Hence, nonstabilizer resources are necessary to achieve universal quantum computation.

In recent literature, the property of not being a stabilizer has been called magic. To quantify the amount of magic, several magic measures have been proposed (52–62), and applied in the classical simulation of quantum circuits (56–61, 63) and unitary synthesis (54, 55). Moreover, to achieve a quantum advantage for DV quantum systems, several sampling tasks have been proposed (64–70). Some of these proposals have been realized in experiment, which were used to claim a computational advantage over classical supercomputers (71–73).

## A. Summary of Main Results.

Little had been known about the extremality of stabilizer states, or their role in the convolution of DV states. We propose a framework to study these questions, based on defining a convolution for DV quantum systems. We explain the intuition behind our approach and state our key results in this paper. The complete details and proofs, as well as a theory of the convolution of quantum channels, appear in an extended, companion work (74).

Our approach is different from the one in refs. (75) and (76). Our convolution of states *ρ* ⊠ *σ* depends on a chosen Clifford unitary, along with a partial trace. We study our approach, with the special goal to reveal extremality of stabilizer states in relation to the convolution. This work includes the following:

1. We introduce the notion of a mean state (MS), which is the closest state in the set of minimal stabilizer-projection states (MSPS) with respect to the relative entropy in Definition 3. We prove the extremality of MSPS: Within all quantum states having the same MS up to Clifford conjugation, the MSPS attains the maximal Rényi entropy. One implication of the extremality of the MS is that it provides a nontrivial, resource-destroying map in the resource theory of magic; Corollary 5.
2. We introduce the notion of the magic gap, which is the difference between the first and second largest absolute values in the support of the characteristic function in Definition 6. We prove that the magic gap can serve as a magic measure; it provides a lower bound on the number of the non-Clifford gates in the synthesis of the unitary. We formulate these results in Propositions 7 and 8.
3. We introduce our convolution ⊠ in Definition 10. A fundamental property is Proposition 12, showing that stabilizer states are closed under convolution. Convolution also increases the generalized quantum Rényi entropy, as stated in Theorem 14. Convolution decreases the Fisher information, as stated in Theorem 15. We state in Theorem 16 that the convolutional channel achieves minimal output entropy, if and only if the input states are pure stabilizer states. We study the Holevo channel capacity of the convolutional channel, and show that the convolutional channel achieves the maximal Holevo capacity if and only if the state is a stabilizer, see Theorem 19.
4. Our convolutional approach includes two important examples, the DV beam splitter and the DV amplifier, both of which share a similar structure to their CV counterparts. We compare our DV results on the beam splitter to the known results for CV quantum systems in Section D, Table 2. We also compare CV and DV cases for the amplifier in Section D, Table 3.
5. We establish a quantum central limit theorem for finite-dimensional quantum systems, based on our discrete convolution, Theorem 24. We also find a “second law of thermodynamics for quantum convolution,” Proposition 22. This means that quantum Rényi entropy *H*_*α*_(⊠^*N*^*ρ*) is nondecreasing with respect to the number *N* of convolutions. Moreover, the repeated convolution of any zero-mean quantum state converges to the MS, with an exponential rate of convergence that is bounded by the magic gap of the state, all stated precisely in Theorem 24.

In the case of CV quantum systems, central limit theorems have an interesting history that goes back to Cushen and Hudson (77), and related work of Hepp and Lieb (78, 79). Many other quantum or noncommutative versions of the central limit theorem appeared later, refs. (80–95).

For example, in free probability theory Voiculescu introduced and studied free convolution and proved a free central limit theorem: the repeated, normalized (additive) free convolution of a probability measure (with some assumptions) converges to a semicircle distribution (96–99). The semicircle distribution in free probability plays a role similar to the Gaussian distribution in classical probability theory.

Several additional central limit theorems have been established in other frameworks. These include results for subfactor theory (90, 91), for quantum walks on a lattice (95), and for CV quantum information theory (93, 94).

## 1. Preliminaries

We focus on the *n*-qudit system ℋ^⊗*n*^, where ℋ ≃ ℂ^*d*^ is a *d*-dimensional Hilbert space and *d* is any natural number. Let *D*(ℋ^⊗*n*^) denote the set of all quantum states on ℋ^⊗*n*^. In the Hilbert space ℋ, we consider the orthonormal, computational basis {|*k*⟩}_*k* ∈ ℤ_*d*__. The Pauli *X* and *Z* operators are

$$X:|k\rangle \mapsto \left|k+1\rangle ,\;\;\;Z:|k\right\rangle \mapsto \xi_{d}^{k}\left|k\right\rangle ,\;\;\;\forall k\in Z_{d},$$

where ℤ_*d*_ is the cyclic group over *d*, and *ξ*_*d*_ = exp(2*πi*/*d*) is a *d*-th root of unity. In order to define our quantum convolution, one needs to restrict *d* to be prime. If *d* is an odd prime number, the local Weyl operators (or generalized Pauli operators) are defined as *w*(*p*, *q*)=*ξ*_*d*_^−2^−1^*pq*^ *Z*^*p*^*X*^*q*^. Here, 2^−1^ denotes the inverse $\frac{d+1}{2}$ of 2 in ℤ_*d*_. If *d* = 2, the Weyl operators are defined as *w*(*p*, *q*)=*i*^−*pq*^*Z*^*p*^*X*^*q*^. Weyl operators for general local dimension *d* are given in ref. (100). In the *n*-qudit system, the Weyl operators are defined as

$$\begin{array}{c} w\left(\vec{p},\vec{q}\right)=w\left(p_{1},q_{1}\right)⊗...⊗w\left(p_{n},q_{n}\right), \end{array}$$

with $\vec{p}=\left(p_{1},p_{2},...,p_{n}\right)\in Z_{d}^{n}$, $\vec{q}=\left(q_{1},...,q_{n}\right)\in Z_{d}^{n}$, which forms an orthonormal basis with respect to the inner product $\left\langle A,B\right\rangle =\frac{1}{d^{n}}\;\text{Tr}\left\{A^{†}B\right\}$. Denote *V*^*n*^ := ℤ_*d*_^*n*^ × ℤ_*d*_^*n*^; this represents the phase space for *n*-qudit systems (49).

Definition 1: For any *n*-qudit state *ρ*, its characteristic function *Ξ*_*ρ*_ : *V*^*n*^ → ℂ is

$$\begin{array}{c} \Xi_{\rho }\left(\vec{p},\vec{q}\right):=\mathrm{Tr}\left\{\rho w(-\vec{p},-\vec{q})\right\}. \end{array}$$

Hence, any quantum state *ρ* can be written as a linear combination of the Weyl operators

[1] $$\begin{array}{c} \rho =\frac{1}{d^{n}}\sum_{\left(\vec{p},\vec{q}\right)\in V^{n}}\Xi_{\rho }\left(\vec{p},\vec{q}\right)w\left(\vec{p},\vec{q}\right). \end{array}$$

The process of taking characteristic functions is the quantum Fourier transform that we consider. The characteristic function has been used to study quantum Boolean functions (101). See also a more general framework of quantum Fourier analysis (102). The Clifford unitaries on *n* qudits are the unitaries that map Weyl operators to Weyl operators. Pure stabilizer states are pure states of the form *U*|0⟩^⊗*n*^, where *U* is some Clifford unitary. Equivalently, pure stabilizer states are the common eigenstates of an abelian subgroup of the Weyl operators with size *d*^*n*^. In general, let us consider any abelian subgroup of the Weyl operators with *r*(≤*n*) generators ${\left\{w\left({\vec{p}}_{i},{\vec{q}}_{i}\right)\right\}}_{i\in [r]}$, and [*r*] denotes the set {0, 1, 2, …, *r*}.

Definition 2: A quantum state *ρ* is a minimal stabilizer-projection state (MSPS) associated with an abelian subgroup generated by ${\left\{w\left({\vec{p}}_{i},{\vec{q}}_{i}\right)\right\}}_{i\in [r]}$, if it has the following form

$$\begin{array}{c} \rho =\frac{1}{d^{n-r}}\Pi_{i=1}^{r}E_{k_{i}\in Z_{d}}{\left[\xi_{d}^{x_{i}}w\left({\vec{p}}_{i},{\vec{q}}_{i}\right)\right]}^{k_{i}}, \end{array}$$

for some (*x*_1_, …, *x*_*r*_)∈ℤ_*d*_^*r*^, with $E_{k_{i}\in Z_{d}}\left(\;\cdot \;\right):=\frac{1}{d}\sum_{k_{i}\in Z_{d}}\left(\;\cdot \;\right)$.

An equivalent, alternative definition is provided in the companion paper (74). Let us consider an example with the abelian group *S* = {*Z*_1_, …, *Z*_*n* − 1_} for an *n*-qudit system. The states ${\left\{\frac{1}{d}|\vec{j}\rangle \;\langle \vec{j}|⊗I\right\}}_{\vec{j}\in Z_{d}^{n-1}}$ are MSPS. Moreover, a quantum state *ρ* is called a stabilizer state if it can be written as a convex combination of pure stabilizer states.

## 2. Mean State

In this section, we introduce the notion of mean state for a given quantum state.

Definition 3 [Mean state (MS)]: Given an *n*-qudit state *ρ*, the mean state ℳ(*ρ*) is the operator with the characteristic function:

[2] $$\begin{array}{c} \Xi_{M(\rho )}\left(\vec{p},\vec{q}\right):=\left\{\begin{array}{ccc} & \Xi_{\rho }\left(\vec{p},\vec{q}\right), & |\Xi_{\rho }\left(\vec{p},\vec{q}\right)|=1,\\ & 0, & |\Xi_{\rho }\left(\vec{p},\vec{q}\right)|<1. \end{array}\right. \end{array}$$

The mean state ℳ(*ρ*) is an MSPS.

We call ℳ(*ρ*) the mean state because we use it to define the mean-value vector of the state *ρ* in Eq. **22** and the zero-mean state in Definition 23. Moreover, we find that the MS is the closest MSPS in quantum Rényi relative entropy *D*_*α*_, where

$$\begin{array}{c} D_{\alpha }\left(\rho ||\sigma \right):=\frac{1}{\alpha -1}\log\mathrm{Tr}\left\{{\left(\sigma^{\frac{1-\alpha }{2\alpha }}\rho \sigma^{\frac{1-\alpha }{2\alpha }}\right)}^{\alpha }\right\}, \end{array}$$

and the quantum Rényi entropy is

$$\begin{array}{c} H_{\alpha }\left(\rho \right):=\frac{1}{1-\alpha }\log\mathrm{Tr}\left\{\rho^{\alpha }\right\}, \end{array}$$

for any *α* ∈ [0, +∞]. For example, the relative entropy *D*(*ρ*||*σ*)=lim_*α* → 1_*D*_*α*_(*ρ*||*σ*), and the von Neumann entropy *H*(*ρ*)=lim_*α* → 1_*H*_*α*_(*ρ*).

Theorem 4 (Extremality of MSPS). *Given an *n*-qudit state *ρ* and *α* ∈ [1, +∞], one has*

$$\begin{array}{c} \min_{\sigma \in MSPS}D_{\alpha }\left(\rho ||\sigma \right)=D_{\alpha }\left(\rho ||M\left(\rho \right)\right)=H_{\alpha }\left(M\left(\rho \right)\right)-H_{\alpha }\left(\rho \right). \end{array}$$

*Moreover, ℳ(*ρ*) is the unique minimizer, i.e., for any *σ* ∈ *MSPS* with *σ* ≠ ℳ(*ρ*), we have*

$$\begin{array}{c} D_{\alpha }\left(\rho ||\sigma \right)>D_{\alpha }\left(\rho ||M\left(\rho \right)\right). \end{array}$$

Based on the above result, we can rewrite the quantum Rényi entropy as follows

[3] $$\begin{array}{c} H_{\alpha }\left(M\left(\rho \right)\right)=H_{\alpha }\left(\rho \right)+D_{\alpha }\left(\rho ||M\left(\rho \right)\right). \end{array}$$

This equation shows the extremality of MSPS with respect to quantum Rényi entropy: Within all quantum states having the same MS up to Clifford conjugation, the MSPS ℳ(*ρ*) attains the maximal value for quantum Rényi entropy, which we call “maximal entropy principle in DV systems.” Recall the extremality of Gaussian states in CV systems, i.e., within all states having a given covariance matrix, Gaussian states attain the maximum von Neumann entropy (2, 3). Hence, the above theorem is the discrete version of the extremality of Gaussian states with the same covariance matrix in CV systems.

In this work, we consider extremality properties of stabilizer states for quantum entropy. One can also consider the classical representation of quantum states, for example by studying the characteristic functions. One entropic measure, such as the 0-Rényi-quantum-Fourier entropy of a pure state *ρ* (defined as the logarithm of the Pauli rank *R*_*P*_(*ρ*) = |Supp(*Ξ*_*ρ*_)|) also achieves its minimal value, iff *ρ* is a stabilizer state (60). Other literature also touches on classical descriptions of quantum states; for example extremality of pure coherent states in the Wehrl entropy is known, as are some variants (20, 103–105).

Corollary 5. *In the resource theory of magic with *MSPS* being the set of free states, the map from quantum states to *MSPS*, namely *ρ* → ℳ(*ρ*), provides a nontrivial, resource-destroying map.*

Note that a map *λ* from states to states is called a resource-destroying map (106) if it satisfies two conditions: i) it maps all quantum states to free states, i.e., *λ*(*ρ*)∈ℱ for any quantum state *ρ*, where ℱ is the set of free states; ii) it preserves free states, i.e., *λ*(*σ*) = *σ* for any state *σ* ∈ ℱ. The natural resource-destroying maps are known in resource theories such as coherence, asymmetry, and non-Gaussianity (Table 1). However, it was unknown what a nontrivial, resource-destroying map is in the resource theory of magic. Here, our work shows that, the map ℳ : 𝒟(ℋ^⊗*n*^)→*MSPS* is a resource-destroying map, which satisfies min_*σ* ∈ *MSPS*_*D*_*α*_(*ρ*||*σ*)=*D*_*α*_(*ρ*||ℳ(*ρ*)).

**Table 1.** Resource theories with a nontrivial, resource-destroying map

Theory	Resource-destroying map
Coherence	*Δ*(*ρ*)=∑_*i*_⟨*i*|*ρ*|*i*⟩|*i*⟩​⟨*i*|, where *Δ* is the complete dephasing channel:{|*i*⟩} w.r.t. the reference basis (107, 108)
Asymmetry	𝒢(*ρ*)=∫_*G*_*dμ*(*U*)*UρU*^†^, where the integral is taken over the Haar measure on *G* (109)
Non-Gaussianity	*λ*(*ρ*)=*ρ*_*G*_, where *ρ*_*G*_ is the Gaussian state with the same mean displacement and covariance matrix as *ρ* (110)
Magic	ℳ(*ρ*), the closest MSPS (Theorem 4 in this work)

Since every quantum state *ρ* can be written as a linear combination of the Weyl operators together with the characteristic function *Ξ*_*ρ*_, the information of the state is encoded in the characteristic function. We consider the gap between the largest absolute value, namely 1, and the second-largest absolute value in the support of the characteristic function. We call this the magic gap (or nonstabilizer gap).

Definition 6 (Magic gap): Given an *n*-qudit state *ρ* ∈ 𝒟(ℋ^⊗*n*^) for any integer *d* ≥ 2, the magic gap of *ρ* is

$$\begin{array}{c} MG\left(\rho \right)=1-\max_{\left(\vec{p},\vec{q}\right)\in \;\text{Supp}\left(\Xi_{\rho }\right):\left|\Xi_{\rho }\left(\vec{p},\vec{q}\right)\right|\neq 1}\left|\Xi_{\rho }\left(\vec{p},\vec{q}\right)\right|. \end{array}$$

If $\{\left(\vec{p},\vec{q}\right)\in \;\text{Supp}\left(\Xi_{\rho }\right):|\Xi_{\rho }\left(\vec{p},\vec{q}\right)|\neq 1\}=\emptyset$, define *MG*(*ρ*)=0, i.e., there is no gap on the support of the characteristic function.

Proposition 7. The magic gap (MG) of a state *ρ* satisfies the following properties:

1. The *MG*(*ρ*)=0, iff *ρ* is an MSPS. Also $0\leq MG\left(\rho \right)\leq 1-\sqrt{\frac{d^{n}\;\text{Tr}\left\{\rho^{2}\right\}-d^{k}}{R_{P}\left(\rho \right)-d^{k}}}$, where *R*_*P*_(*ρ*)=|Supp(*Ξ*_*ρ*_)| is the Pauli rank (60).
2. The *MG* is invariant under Clifford unitaries.
3. *MG*(*ρ*_1_ ⊗ *ρ*_2_)=min{*MG*(*ρ*_1_),*MG*(*ρ*_2_)}.

Since −log(1 − *x*)=*x* + *O*(*x*^2^), we can also consider the logarithmic magic gap (LMG), that is,

$$\begin{array}{c} LMG(\rho )=-\log\underset{(\vec{p},\vec{q})\in \;\text{Supp}({\text{Ξ}}_{\rho }):|{\text{Ξ}}_{\rho }(\vec{p},\vec{q})|\neq 1}{\max}|{\text{Ξ}}_{\rho }(\vec{p},\vec{q})|. \end{array}$$

This *LMG*(*ρ*) also satisfies conditions (1–3) in Proposition 7 by changing the upper bound in (1) to $\frac{1}{2}\log\left[\frac{R_{P}\left(\rho \right)-d^{k}}{d^{n}\;\text{Tr}\left\{\rho^{2}\right\}-d^{k}}\right]$.

Now, let us consider the application of the magic gap in the unitary synthesis. In an *n*-qubit system, the universal quantum circuits consist of Clifford gates and *T* gates. From the Gottesman–Knill theorem (50), we infer that Clifford unitaries can be simulated efficiently on a classical computer. So the *T* gates (or other non-Clifford gates) are the source of any quantum computational advantage. Hence, it is important to determine how many *T* gates are necessary to generate the target unitary. We find that the logarithm of the magic gap can provide a lower bound on the number of *T* gates.

Proposition 8. *Given an input state *ρ* and a quantum circuit *V*_*N*_, consisting of Clifford unitaries and *N* magic T gates, the log magic gap of the output state *V*_*N*_*ρV*_*N*_^†^ satisfies,*

$$\begin{array}{c} LMG\left(V_{N}\rho V_{N}^{†}\right)\leq LMG\left(\rho \right)+\frac{N}{2}. \end{array}$$

## 3. Convolution in DV Quantum Systems

We introduce the convolution between 2 different *n*-qudit systems, denoted by ℋ_*A*_ and ℋ_*B*_, respectively. In other words, the Hilbert spaces are ℋ_*A*_ = ℋ^⊗*n*^, and ℋ_*B*_ = ℋ^⊗*n*^, where dim ℋ = *d*.

### A. Discrete Convolution.

Given a prime number *d*, consider the 2 × 2 invertible matrix of parameters,

[4] $$\begin{array}{c} G=\left[\begin{array}{cc} g_{00} & g_{01}\\ g_{10} & g_{11} \end{array}\right]:=\left[g_{00},g_{01};g_{10},g_{11}\right], \end{array}$$

with entries in ℤ_*d*_. We assume that *G* is invertible in ℤ_*d*_, so $\det\;G=g_{00}g_{11}-g_{01}g_{10}≢0\;\mathrm{mod}\;d$. The inverse in ℤ_*d*_ is

[5] $$\begin{array}{c} G^{-1}=N\left[\begin{array}{cc} g_{11} & -g_{01}\\ -g_{10} & g_{00} \end{array}\right],\;\;\text{where}N={\left(\det\;G\right)}^{-1}. \end{array}$$

The matrix *G* is called positive if none of *g*_*ij*_ ≡ 0mod*d*. In this work, we focus on the case where *G* is positive and invertible. If *d* is an odd prime number, there always exists a positive and invertible matrix *G* in ℤ_*d*_, e.g., *G* = [1, 1; 1, *d* − 1]. If *d* = 2, there is no positive and invertible matrix *G*, as the only positive matrix [1, 1; 1, 1] is not invertible in ℤ_2_.

Definition 9 (Key unitary): Given a positive and invertible matrix *G*, a 2*n*-qudit unitary *U* is

[6] $$\begin{array}{c} U=\underset{\vec{i},\vec{j}}{\sum }|{\vec{i}}^{\prime }\rangle \langle \vec{i}|⊗|{\vec{j}}^{\prime }\rangle \langle \vec{j}|, \end{array}$$

where the state $|\vec{i}\rangle =|i_{1}\rangle ⊗\cdots ⊗|i_{n}\rangle \in H^{⊗n}$, and $\left[\begin{array}{c} i\prime_{k}\\ j\prime_{k} \end{array}\right]={\left(G^{-1}\right)}^{T}\left[\begin{array}{c} i_{k}\\ j_{k} \end{array}\right]$, for *k* ∈ [*n*].

That is, *U* maps the state $|\vec{i},\vec{j}\rangle$ to the state $\left|Ng_{11}\vec{i}\;-\right.$$Ng_{10}\vec{j},-Ng_{01}\vec{i}+Ng_{00}\vec{j}\rangle$, where *N* = (*det* *G*)^−1^ = (*g*_00_*g*_11_ − *g*_01_*g*_10_)^−1^.

Definition 10 (Convolution of states): Given the Clifford unitary *U* in (**6**), and two quantum states *ρ* ∈ 𝒟(ℋ_*A*_),*σ* ∈ 𝒟(ℋ_*B*_), the convolution of *ρ* and *σ* is

[7] $$\begin{array}{c} \rho ⊠\sigma ={\mathrm{Tr}}_{B}\left\{U\left(\rho ⊗\sigma \right)U^{†}\right\}. \end{array}$$

The partial trace is taken on the second *n*-qudit system ℋ_*B*_. The corresponding quantum convolutional channel ℰ is

[8] $$\begin{array}{c} E\left(\rho_{\mathrm{AB}}\right)={\mathrm{Tr}}_{B}\left\{U\left(\rho_{\mathrm{AB}}\right)U^{†}\right\}, \end{array}$$

for any quantum state *ρ*_*AB*_ on ℋ_*A*_ ⊗ ℋ_*B*_.

Proposition 11 (Convolution-multiplication duality). Given the convolution with the parameter matrix *G*, the characteristic function satisfies

$$\begin{array}{c} \Xi_{\rho ⊠\sigma }\left(\vec{p},\vec{q}\right)=\Xi_{\rho }\left(Ng_{11}\vec{p},g_{00}\vec{q}\right)\;\Xi_{\sigma }\left(-Ng_{10}\vec{p},g_{01}\vec{q}\right), \end{array}$$

for any $\vec{p},\vec{q}\in Z_{d}^{n}$.

In classical probability theory, the convolution of two Gaussian distributions is still a Gaussian. Here, we find the analogous property for stabilizer states.

Proposition 12 (Convolutional stability). *Given two *n*-qudit stabilizer states *ρ* and *σ*, *ρ* ⊠ *σ* is a stabilizer state.*

It is well-known that the distance measure is monotone under the convolution in classical probability theory,

$$D\left(\mu_{1}*\nu ,\mu_{2}*\nu \right)\leq D\left(\mu_{1},\mu_{2}\right),$$

for measures *μ*_1_, *μ*_2_, *ν* on ℝ^*d*^, where *D* is either the classical total variation distance, relative divergence, or Wasserstein distance. Here, we establish a quantum version of the monotonicity of distance measures under quantum convolution, for the distance measures including the *L*_1_ norm, relative entropy, and quantum Wasserstein distance (defined in ref. (111)).

Proposition 13 (Monotonicity under convolution). *Let the distance measure *D* : 𝒟(ℋ^⊗*n*^)×𝒟(ℋ^⊗*n*^)→ℝ be the *L*_1_ norm, relative entropy, or quantum Wasserstein distance. Then, for any convolution ⊠ with respect to the positive and invertible matrix *G*, we have*

[9] $$\begin{array}{c} D(\rho ⊠\tau ,\sigma ⊠\tau )\leq D(\rho ,\sigma ). \end{array}$$

### B. Quantum Entropy and Fisher-Information Inequalities.

Consider the behavior of the generalized quantum Rényi entropy (112) under convolution. Here,

[10] $$\begin{array}{c} H_{\alpha }\left(\rho \right):=\frac{\mathrm{sgn}(\alpha )}{1-\alpha }\log\sum_{i}\lambda_{i}^{\alpha },\;\forall \alpha \in \left[-\infty ,+\infty \right], \end{array}$$

where *λ*_*i*_ are the eigenvalues of *ρ*, and sgn(*α*)= ± 1.

Theorem 14 (Convolution increases Rényi entropy). *Let the parameter matrix *G* be positive and invertible, and *ρ*, *σ* be two *n*-qudit states. The generalized Rényi entropy satisfies*

[11] $$\begin{array}{c} H_{\alpha }\left(\rho ⊠\sigma \right)\geq \max\left\{H_{\alpha }\left(\rho \right),H_{\alpha }\left(\sigma \right)\right\}, \end{array}$$

for any *α* ∈ [ − ∞, +∞].

Besides quantum Rényi entropies, we also consider the divergence-based quantum Fisher information (9): Given a smooth one-parameter family of states {*ρ*_*θ*_}_*θ*_, the divergence-based quantum Fisher information at 0 is defined as

$$\begin{array}{c} J\left(\rho^{\theta };\theta \right){|}_{\theta =0}:=\frac{d^{2}}{d\theta^{2}}{|}_{\theta =0}D(\rho ||\rho_{\theta }). \end{array}$$

Since the first derivative $\frac{d}{d\theta }{|}_{\theta =0}D(\rho ||\rho_{\theta })=0$, the second derivative *J*(*ρ*^*θ*^; *θ*)|_*θ* = 0_ quantifies the sensitivity of the divergence with respect to the change of parameter *θ*. Since we only consider the divergence-based quantum Fisher information *J*(*ρ*^*θ*^; *θ*)|_*θ* = 0_ in this work, we call it the quantum Fisher information for simplicity. If {*ρ*_*θ*_}_*θ*_ is a family of parameterized states defined by *ρ*_*θ*_ = exp(*iθH*)*ρ*exp(−*iθH*) with respect to a Hermitian operator *H* for all *θ* ∈ ℝ, then the quantum Fisher information can be written as

$$\begin{array}{c} J\left(\rho ;H\right)=\frac{d^{2}}{d\theta^{2}}{|}_{\theta =0}D(\rho ||\rho_{\theta })=\mathrm{Tr}\left\{\rho [H,[H,\log\rho ]]\right\}. \end{array}$$

In *n*-qudit systems, we denote *X*_*k*_ (resp., *Z*_*k*_) to be the Pauli *X* (resp., *Z*) operator on *k*-th qudit. For *R* = *X*_*k*_ or *Z*_*k*_ (1 ≤ *k* ≤ *n*), denote |*j*⟩_*R*_ to be an eigenvector of *R* corresponding to the eigenvalue *ξ*_*d*_^*j*^ with *j* ∈ ℤ_*d*_. Let us define the Hermitian operator *H*_*j*_^*R*^ for *j* ∈ [*d*] as *H*_*j*_^*R*^ = |*j*⟩​⟨*j*|_*R*_, and the corresponding parameterized unitary *U*_*j*_^*R*^(*θ*) as *U*_*j*_^*R*^(*θ*)=exp(*iθH*_*j*_^*R*^). Then, for any quantum state *ρ*, let us consider the family of parameterized states *ρ*_*R*, *θ*_ = *U*_*j*_^*R*^(*θ*)*ρU*_*j*_^*R*^(*θ*)^†^, *θ* ∈ ℝ, and the corresponding quantum Fisher information *J*(*ρ*; *H*_*j*_^*R*^). Let us denote

[12] $$\begin{array}{c} J\left(\rho \right)=\sum_{k=1}^{n}\sum_{j=1}^{d}J\left(\rho ;H_{j}^{X_{k}}\right)+J\left(\rho ;H_{j}^{Z_{k}}\right). \end{array}$$

Theorem 15 (Convolution decreases Fisher information). *Let the parameter matrix *G* be positive and invertible, and *ρ*, *σ* be two *n*-qudit states. The quantum Fisher information satisfies*

[13] $$\begin{array}{c} J(\rho ⊠\sigma )\leq \min\{J(\rho ),J(\sigma )\}. \end{array}$$

### C. Stabilizer States in the Convolutional Channel.

What kind of input states *ρ*, *σ* will make the output state have the minimal output entropy?

Theorem 16. *Let the parameter matrix *G* be positive and invertible, and *ρ*, *σ* be two *n*-qudit states. The output state ℰ(*ρ* ⊗ *σ*) has the minimal output entropy iff both *ρ* and *σ* are pure stabilizer states, and the stabilizer groups *S*_1_ and *S*_2_ of *ρ* and *σ* satisfy*

[14] $$\begin{array}{c} S_{1}=\left\{w\left(-g_{10}^{-1}g_{11}\vec{x},g_{01}^{-1}g_{00}\vec{y}\right):w\left(\vec{x},\vec{y}\right)\in S_{2}\right\}. \end{array}$$

Besides, we consider the Holevo capacity of the quantum channel, which can be used to quantify the classical capacity of a memoryless quantum channel (113, 114).

Definition 17 (Holevo capacity): Given a quantum channel ℰ, the Holevo capacity *χ*(ℰ) is

$$\begin{array}{c} \chi \left(E\right)=\max_{\left\{p_{i},\rho_{i}\right\}}H\left(\sum_{i}p_{i}E\left(\rho_{i}\right)\right)-\sum_{i}p_{i}H\left(E\left(\rho_{i}\right)\right), \end{array}$$

where the maximum is taken over all ensembles {*p*_*i*_, *ρ*_*i*_} of possible input states *ρ*_*i*_ occurring with probabilities *p*_*i*_.

Given a quantum state *σ*, the quantum channel ℰ_*σ*_(⋅)=ℰ(⋅ ⊗ *σ*). That is, for any input state *ρ*, the output state of the channel ℰ_*σ*_ is *ρ* ⊠ *σ*. We find that the Holevo capacity of ℰ_*σ*_ can be bounded by the entropies of both *σ* and ℳ(*σ*).

Theorem 18 (Holevo capacity bound: general case). *Let the parameter matrix *G* be positive and invertible, and *σ* be an *n*-qudit state. The Holevo capacity of the quantum channel ℰ_*σ*_ is*

[15] $$\begin{array}{c} n\log d-H\left(M\left(\sigma \right)\right)\leq \chi \left(E_{\sigma }\right)\leq n\log d-H\left(\sigma \right). \end{array}$$

If *σ* ∈ *MSPS*, then

[16] $$\begin{array}{c} \chi \left(E_{\sigma }\right)=n\log d-H\left(\sigma \right). \end{array}$$

Besides, we find that the pure stabilizer states are the only states making the convolutional channel ℰ_*σ*_ achieve the maximal Holevo capacity.

Theorem 19 (Maximizer for Holevo capacity: pure stabilizer states). Let the parameter matrix *G* be positive and invertible. The quantum channel ℰ_*σ*_ has the maximal Holevo capacity *n*log*d* iff *σ* is a pure stabilizer state.

### D. Examples: Discrete Beam Splitter and Amplifier.

Now, let us consider two examples of convolutions. The first one is the discrete beam splitter with *G* = [*s*, *t*; *t*, −*s*] and *s*^2^ + *t*^2^ ≡ 1mod*d*. This is a discrete version of the condition ${\left(\sqrt{\lambda }\right)}^{2}+{\left(\sqrt{1-\lambda }\right)}^{2}=1$ that occurs in CV beam splitter. In fact, the condition *s*^2^ + *t*^2^ ≡ 1mod*d* can be satisfied for any prime number *d* ≥ 7 by some number theory guarantee. Formally, we have the following definition of discrete splitter beam.

Definition 20 (Discrete beam splitter): *Given *s*^2^ + *t*^2^ ≡ 1mod*d*, the unitary operator *U*_*s*, *t*_ is*

[17] $$\begin{array}{c} U_{s,t}=\sum_{\vec{i},\vec{j}\in Z_{d}^{n}}|s\vec{i}+t\vec{j}\rangle \langle \vec{i}|⊗|t\vec{i}-s\vec{j}\rangle \langle \vec{j}|, \end{array}$$

where the state$|\vec{i}\rangle =|i_{1}\rangle ⊗\cdots ⊗|i_{n}\rangle \in H^{⊗n}$. The convolution of two *n*-qudit states *ρ* and *σ* is

[18] $$\begin{array}{c} \rho {⊠}_{s,t}\sigma ={\mathrm{Tr}}_{B}\left\{U_{s,t}\left(\rho ⊗\sigma \right)U_{s,t}^{†}\right\}. \end{array}$$

We summarize and compare our results on discrete beam splitter with the known results for CV quantum systems in Table 2.

**Table 2.** Comparison of results for the CV and DV beam splitters

Beam splitter	CV quantum systems	DV quantum systems
Parameter	$\left(\sqrt{\lambda },\sqrt{1-\lambda }\right),\lambda \in \left[0,1\right]$	(*s*, *t*), *s*^2^ + *t*^2^ ≡ 1mod*d*
Convolution	*ρ*⊠_*λ*_*σ* = Tr_*B*_{*U*_*λ*_*ρ*⊗*σU*_*λ*_^†^},	*ρ*⊠_*s*, *t*_*σ* = Tr_*B*_{*U*_*s*, *t*_*ρ*⊗*σU*_*s*, *t*_^†^},
	*U*_*λ*_: beam splitter	*U*_*s*, *t*_ : discrete beam splitter
Characteristic function	$\Xi_{\rho {⊠}_{\lambda }\sigma }\left(\vec{x}\right)=\Xi_{\rho }\left(\sqrt{\lambda }\vec{x}\right)\Xi_{\sigma }\left(\sqrt{1-\lambda }\vec{x}\right)$	$\Xi_{\rho {⊠}_{s,t}\sigma }\left(\vec{x}\right)=\Xi_{\rho }\left(s\vec{x}\right)\Xi_{\sigma }\left(t\vec{x}\right)$
Quantum entropy inequality	*H*(*ρ*⊠_*λ*_*σ*)≥*λH*(*ρ*)+(1 − *λ*)*H*(*σ*) (9)	*H*_*α*_(*ρ*⊠_*s*, *t*_*σ*)≥max{*H*_*α*_(*ρ*),*H*_*α*_(*σ*)},
	*e*^*H*(*ρ*⊠_*λ*_*σ*)/*n*^ ≥ *λe*^*H*(*ρ*)/*n*^ + (1 − *λ*)*e*^*H*(*σ*)/*n*^ (9, 10)	*α* ∈ [ − ∞, ∞](Theorem 14)
Quantum Fisher information inequality	*w*^2^*J*(*ρ*⊠_*λ*_*σ*)≤*w*_1_^2^*J*(*ρ*)+*w*_2_^2^*J*(*σ*),	*J*(*ρ*⊠_*s*, *t*_*σ*)≤min{*J*(*ρ*),*J*(*σ*)}
	$w=\sqrt{\lambda }w_{1}+\sqrt{1-\lambda }w_{2}$ (9)	(Theorem 15)

Besides the discrete beam splitter, we define the discrete amplifier with *G* = [*l*, −*m*; −*m*, *l*] and *l*^2^ − *m*^2^ ≡ 1mod*d*. This is a discrete version of the condition ${\left(\sqrt{\kappa }\right)}^{2}+{\left(\sqrt{\kappa -1}\right)}^{2}=1$ with *κ* ∈ [1, ∞] that occurs in CV squeezing unitary. In fact, the condition *l*^2^ − *m*^2^ ≡ 1mod*d* can also be satisfied for any prime number *d* ≥ 7 by some number theory guarantee. Formally, we have the following definition of discrete amplifier.

Definition 21 (Discrete amplifier): Given *l*^2^ − *m*^2^ ≡ 1mod*d*, the unitary operator *V*_*l*, *m*_ is

[19] $$\begin{array}{c} V_{l,m}=\sum_{\vec{i},\vec{j}\in Z_{d}^{n}}|l\vec{i}+m\vec{j}\rangle \langle \vec{i}|⊗|m\vec{i}+l\vec{j}\rangle \langle \vec{j}|. \end{array}$$

The convolution of two *n*-qudit states *ρ* and *σ* is

[20] $$\begin{array}{c} \rho {⊠}_{l,m}\sigma ={\mathrm{Tr}}_{B}\left\{V_{l,m}\left(\rho ⊗\sigma \right)V_{l,m}^{†}\right\}. \end{array}$$

We summarize and compare our results on discrete amplifier with the known results for CV quantum systems in Table 3.

**Table 3.** Comparison of results for the CV and DV amplifiers

Amplifier	CV quantum systems	DV quantum systems
Parameter	$\left(\sqrt{\kappa },\sqrt{\kappa -1}\right),\kappa \in \left[1,\infty \right]$	(*l*, *m*), *l*^2^ − *m*^2^ ≡ 1mod*d*
Convolution	*ρ*⊠_*κ*_*σ* = Tr_*B*_{*V*_*κ*_*ρ*⊗*σV*_*κ*_^†^},	*ρ*⊠_*l*, *m*_*σ* = Tr_*B*_{*V*_*l*, *m*_*ρ*⊗*σV*_*l*, *m*_^†^},
	*V*_*κ*_ : squeezing unitary	*V*_*l*, *m*_ : discrete squeezing unitary
Characteristic function	$\Xi_{\rho {⊠}_{\kappa }\sigma }\left(\vec{p},\vec{q}\right)=\Xi_{\rho }\left(\sqrt{\kappa }\vec{p},\sqrt{\kappa }\vec{q}\right)\;\Xi_{\sigma }\left(\sqrt{\kappa -1}\vec{p},-\sqrt{\kappa -1}\vec{q}\right)$	$\Xi_{\rho {⊠}_{l,m}\sigma }\left(\vec{p},\vec{q}\right)=\Xi_{\rho }\left(l\vec{p},l\vec{q}\right)\;\Xi_{\sigma }\left(m\vec{p},-m\vec{q}\right)$
Quantum entropy inequality	*e*^*H*(*ρ*⊠_*κ*_*σ*)/*n*^ ≥ *κe*^*H*(*ρ*)/*n*^ + (*κ* − 1)*e*^*H*(*σ*)/*n*^ (10)	*H*_*α*_(*ρ*⊠_*l*, *m*_*σ*)≥max{*H*_*α*_(*ρ*),*H*_*α*_(*σ*)}, *α* ∈ [ − ∞, +∞](Theorem 14)
Quantum Fisher information inequality	*w*^2^*J*(*ρ*⊠_*κ*_*σ*)≤*w*_1_^2^*J*(*ρ*)+*w*_2_^2^*J*(*σ*), $w=\sqrt{\kappa }w_{1}+\sqrt{\kappa -1}w_{2}$ (9)	*J*(*ρ*⊠_*l*, *m*_*σ*)≤min{*J*(*ρ*),*J*(*σ*)} (Theorem 15)

## 4. The Central Limit Theorem

Let us denote that ⊠^*N* + 1^*ρ* = (⊠^*N*^*ρ*)⊠*ρ*, and ⊠^0^*ρ* = *ρ*, where ⊠ is short for the beam splitter convolution ⊠_*s*, *t*_ in **17** (which does not require *s* ≡ *t*mod*d*). By applying Theorem 14, we find that quantum Rényi entropy *H*_*α*_(⊠^*N*^*ρ*) is increasing w.r.t. the number of convolutions *N*.

Proposition 22 (Second law of thermodynamics for quantum convolution). *For any *n*-qudit state *ρ*, the quantum Rényi entropy satisfies the following property,*

[21] $$\begin{array}{c} H_{\alpha }\left({⊠}^{N+1}\rho \right)\geq H_{\alpha }\left({⊠}^{N}\rho \right),\;\forall N\geq 0, \end{array}$$

for any *α* ∈ [ − ∞, +∞].

Note that in the classical case (20, 115, 116), it was proved that $H\left(\frac{X_{1}+X_{2}+...X_{N+1}}{\sqrt{N+1}}\right)\geq H\left(\frac{X_{1}+X_{2}+...X_{N}}{\sqrt{N}}\right)$, where *X*_1_, *X*_2_, … are i.i.d., square-integrable random variables; this is a classical analogue of the second law of thermodynamics.

Before considering the quantum central limit theorem, let us first look at the classical case. Given a random variable *X* with probability density function *f*, if *X* has zero mean, then $\frac{1}{\sqrt{N}}X_{1}+\cdots +\frac{1}{\sqrt{N}}X_{N}$ will converge to some normal random variable, that is, the probability density function *f*_⊠*N*_ converges to a normal distribution as *N* → ∞, where *f*_⊠*N*_ denotes the balanced *N*-th convolution of *f*. The condition that *X* has zero mean cannot be removed. For example, if *X* ∼ 𝒩(1, 1), $\frac{1}{\sqrt{N}}X_{1}+\cdots +\frac{1}{\sqrt{N}}X_{N}∼N\left(\sqrt{N},1\right)$ and does not have a limit distribution. Hence, given a random variable *X*, we should consider the zero-mean variable *X* − 𝔼*X* instead of *X*, where 𝔼*X* denotes the mean value of *X*.

For classical multivariable random variable $\vec{X}\in R^{r}$, its characteristic function is

$$\begin{array}{c} \phi_{X}\left(\vec{t}\right)=E_{\vec{X}}\exp\left(i\vec{t}\cdot \vec{X}\right),\;\forall \vec{t}=\left(t_{1},..,t_{r}\right)\in R^{r}, \end{array}$$

and mean-value vector $\vec{\mu }$ equals to the gradient of $\phi_{X}\left(\vec{t}\right)$ at $\vec{t}=\vec{0}$, i.e.,

$$\begin{array}{c} \vec{\mu }={\left(-i{\left.\frac{d}{dt_{j}}\right|}_{\vec{t}=\vec{0}}\phi_{X}\left(\vec{t}\right)\right)}_{j=1}^{r}. \end{array}$$

If *X* is zero-mean, then $\vec{\mu }=\left(0,...,0\right)$.

For the quantum case, we also need to define the zero-mean state to consider the quantum central limit theorem. Given an *n*-qudit state *ρ*, the MS ℳ(*ρ*) has the characteristic function

$$\begin{array}{c} \Xi_{M(\rho )}\left(\sum_{i=1}^{r}t_{i}\left({\vec{p}}_{i},{\vec{q}}_{i}\right)\right)=\Pi_{i=1}^{r}\xi_{d}^{t_{i}k_{i}}=\xi_{d}^{\sum_{i}t_{i}k_{i}}, \end{array}$$

where we assume the abelian group of ℳ(*ρ*) is generated by the Weyl operators ${\left\{w\left({\vec{p}}_{i},{\vec{q}}_{i}\right)\right\}}_{i\in [r]}$, and $\Xi_{\rho }\left({\vec{p}}_{i},{\vec{q}}_{i}\right)=\xi_{d}^{k_{i}},\forall i\in \left[r\right]$. Similar to the classical case, we define the mean-value vector of the state *ρ* w.r.t. the generators ${\left\{w\left({\vec{p}}_{i},{\vec{q}}_{i}\right)\right\}}_{i\in [r]}$ as

[22] $$\begin{array}{c} {\vec{\mu }}_{M(\rho )}=\left(k_{1},...,k_{r}\right)\;\mathrm{mod}\;d. \end{array}$$

Definition 23 (Zero-mean state): Given an *n*-qudit state *ρ*, *ρ* is called a zero-mean state if ℳ(*ρ*) has mean-value vector ${\vec{\mu }}_{M(\rho )}=\left(0,...,0\right)\;\mathrm{mod}\;d$, or equivalently the characteristic function of ℳ(*ρ*) takes values in {0, 1}.

In fact, if *ρ* is not a zero-mean state, there exists a Weyl operator $w(\vec{p},\vec{q})$ such that $w\left(\vec{p},\vec{q}\right)\rho w{\left(\vec{p},\vec{q}\right)}^{†}$ is a zero-mean state. Now, we have the following result on the quantum central limit theorem for the *L*_2_ norm, where the rate of convergence is controlled by the magic gap.

Theorem 24 (Central limit theorem via magic gap). *Given a zero-mean *n*-qudit state *ρ*, we have*

[23] $$\begin{array}{c} {\left|\left|{⊠}^{N}\rho -M\left(\rho \right)\right|\right|}_{2}\leq {\left(1-MG\left(\rho \right)\right)}^{N}{\left|\left|\rho -M(\rho )\right|\right|}_{2}. \end{array}$$

If *ρ* ≠ ℳ(*ρ*), then *MG*(*ρ*)> 0, and the rate of convergence is exponentially small with respect to the time of convolution.

## 5. Some Open Problems

There are many open questions, such as:

1. Aside from Clifford unitaries, matchgate (117), or Gaussian fermionic operations (118–120) is another tractable family of quantum circuits. Could our convolution be helpful to define matchgates for qudits?
2. In graph theory, the Cheeger constant measures the edge expansion of a graph. The Cheeger inequalities relate the spectral gap of the adjacency matrix of a graph to its Cheeger constant (121–123). Is there a quantum Cheeger constant that corresponds to the magic gap?
3. Can one generalize our convolution using picture languages, such as the Quon language (100), the tensor network (124, 125), the ZX calculus (126, 127), or so on?
4. Following the convolution proposed and studied in refs. (75) and (76), many generalizations have been studied (128, 129), including a generalization to certain von Neumann algebras (129). Will similar generalizations be possible for the convolution in this paper?
5. Similar to the classical case (130, 131), we can explore the entropic limit theorem for quantum convolution. Due to the continuity of relative entropy, *D*(⊠^*N*^*ρ*||ℳ(*ρ*)) converges to 0. Can one determine the rate of convergence?
6. Clarify the relation between the convolution and central limit theorem in this work to their counterparts in free probability theory. The free convolution corresponds to the free independence of random variables. What independence relation corresponds to our convolution?

## Data Availability

There are no data underlying this work.
